# Determining effects of water and nitrogen input on maize (*Zea mays*) yield, water- and nitrogen-use efficiency: A global synthesis

**DOI:** 10.1038/s41598-020-66613-6

**Published:** 2020-06-16

**Authors:** Yuan Li, Song Cui, Zhixin Zhang, Kezhang Zhuang, Zhennan Wang, Qingping Zhang

**Affiliations:** 1 College of Agriculture and Forestry Science, Linyi University, Linyi, 276000 P. R. China; 2 Biogeochemistry Research Group, Department of Environmental and Biological Sciences, University of Eastern Finland, PO Box 1627, Kuopio, Finland; 3 School of Agriculture, Middle Tennessee State University, Murfreesboro, TN 37132 USA; 4 College of Grassland Agriculture, Northwest A&F University, Yangling, 712100 P. R. China; 5 Linyi Agricultural Academy of Sciences, Linyi, Shandong, 276012 P. R. China

**Keywords:** Environmental impact, Plant stress responses

## Abstract

A major challenge in maize (*Zea mays*) production is to achieve high grain yield (yield hereafter) by improving resource use efficiency. Using a dataset synthesized from 83 peer-reviewed articles, this study mainly investigated the effects of water and/or nitrogen (N) input on maize yield, water productivity (WP), and N use efficiency (NUE); and evaluated the effects caused by planting density, environmental (temperature, soil texture), and managerial factors (water and/or N input). The input of water increased maize yield, WP, and NUE only when the input was less than 314, 709, and 311 mm, respectively; input of N increased maize yield, WP, and NUE until input was greater than 250, 128, and 196 kg ha^−1^, respectively. Additionally, results of the mixed-effects model and random forest analysis suggested that mean annual temperature (MAT) was the most critical factor for narrowing gaps (between the actual and attainable variable, which was indicated as response ratio of the treatment relative to the control) of yield (*RR*_Y_), WP (*RR*_WP_), and NUE (*RR*_NUE_), respectively. Specifically, *RR*_Y_, *RR*_WP_, or *RR*_NUE_ were negatively correlated to MAT when MAT was higher than 15 °C. Additionally, the structural equation model showed that water input and *RR*_WP_ with the higher coefficient were more important than N input and *RR*_NUE_ in improving *RR*_Y_. These findings provide new insights into the causes and limitations of global maize production and offer some guidances for water and/or N managements.

## Introduction

Considering the rapidly increasing global population and diminishing water and land resources for supporting agricultural production, mankind faces challenges in food security and maintaining ecological sustainability^1^. Cereal crops are vital sources for sustaining the nutritional/energy need of the global population^2^. Particularly, maize (*Zea mays*) is one of the most important cereal crops for supporting the production of ethanol, livestock feed and others food products including starch and syrup^3^. Consequentially, it is also one of the most commonly grown crops, accounting for 10% of global crop production in the period from 1996 to 2005^4^. Therefore, enhancing maize productivity and resource use efficiency by developing and adopting appropriate managerial practices is important for maintaining global food supply and ecological resilience^2^.

Water and nitrogen (N) have long been recognized as two major limiting input constraints for maize production^5^. Water consumption of main cereal crops was expected to increase by 41% from 2000 to 2015 and given current crop water productivity (WP), there will be an increasing demand due to production and global warming^6^. As a C_4_ plant, maize typically offers superior WP compared with C_3_ crops (e.g. alfalfa (*Medicago sativa*)  or wheat (*Triticum aestivum*)), however, it still consumes greater amount of water due to high biomass production and large evaporation losses during the growing season. Specifically, based on a review of 27 publications across 4 continents, Zwart and Bastiaanssen^7^ summarized that global average WP for maize was 1.8 kg m^−3^ with great variability between 1.1 and 2.7 kg m^−3^. Likewise, the global average N application rate of maize was 80 kg ha^−1^ yr^−1^ from 1961 to 2010^8^, and had resulted in an overall low N use efficiency (NUE, < 33%);^5^. While high water and N saving potentials have also been reported across many global maize production areas^9^. Thus, there is a strong need for conducting quantitative analysis on actual and potential WP and NUE of maize to better understand crucial managerial practices that could affect its yield, WP, and NUE. In particular, presenting such a study across a wide scale to overcome complex interactions with the environment caused by spatial and temporal heterogeneities. Numerous studies have reported the diminishing contributory effects of either water or N on maize yield increase^9,10^, however, the critical response thresholds (i.e. water and N) for productivity and major efficiency indices (i.e. WP and NUE) on a cropping system scale were rarely synthesized and remain poorly understood.

Additionally, temperature is extremely important in affecting maize grain yield and resource use efficiency^11,12^. Temperature affects both the rate and duration of grain growth and influences the persistence and productivity of leaves. For example, maize under an average temperature of 21 °C had greater leaf area duration after silking than that under either 25 °C or 18 °C^13^. Yield modelling using the scenarios of lowest (RCP2.6) to highest (RCP8.5) emissions indicate that maize will suffer greater yield reductions than wheat, rice (*Oryza sativa* L.), or soybean (*Glycine max* L.)^14^ in response to global warming. The effects of temperature can also be confounded with other environmental/managerial factors (e.g. water stress, or radiation intensity). Tollenaar and Wu^15^ reported that increased stress tolerance is associated with lower plant-to-plant variability and high-stress tolerance will probably provide the highest potential for yield improvement in maize. Therefore, advanced data analysis methods and factorization techniques are warranted to better understand the role of temperature in regulating the effect of water or N inputs on maize yield, WP, and NUE. To our knowledge, there have been limited attempts to characterize gaps of maize yield, WP, and NUE response to water, and N inputs, and their associated effects on aspects related to temperature.

In addition, based on a previous study using a meta-analysis approach, we identified large gaps between the actual and attainable yield, WP, and NUE on maize production^9^. Here, we defined the “gap” as the difference between the observed yield, WP, or NUE (actual value) in a given year of a study, and the highest reported yield, WP, or NUE (attainable value) in a given year of a study^9,16^. Therefore, this study aimed to: i) identify a series of important ecophysiological thresholds of water and/or N inputs on optimizing maize yield, WUE, and NUE; ii) summarize the actual and attainable yield, WP, and NUE in the main maize producing countries; and iii) assess the roles of environmental and management factors on affecting maize yield and resource use efficiency.

## Results

### Overview of maize yield, WP and NUE

Figure S1 displays maize yield, WP, and NUE across the main maize production areas in the world. The overall mean yield (ranging from 4.6 to 12 t ha^−1^) was higher than that yield in most countries with exceptions of China (mean ± standard deviation, 8.8 ± 3.3 t ha^−1^, median = 8.6, n = 800), Croatia (9.6 ± 2.7 t ha^−1^, median = 10.3, n = 36), Spain (11.1 ± 1.6 t ha^−1^, median = 11.1, n = 4), and USA (11.1 ± 3.0 t ha^−1^, median = 11.8, n = 240) (Fig. S2a). Water productivity ranged from 23 to 1077 kg  m^−^^3^ for all observations (Fig. S2b). WP in China (1.9 ± 0.9 kg m^−3^, median = 1.8, n = 815), Croatia (1.8 ± 0.6 kg m^−3^, median = 1.8, n = 36), Turkey (3.5 ± 0.8 kg m^−3^, median = 3.9, n = 6), and USA (2.3 ± 0.8 kg m^−3^, median = 2.3, n = 243) were greater than the overall mean WP and all other countries. NUE varied widely (Fig. S2c), NUE in Argentina (100.2 ± 51.3 kg kg^−1^, median = 91.5, n = 16), Brazil (71.9 ± 34 kg kg^−1^, median = 62.5, n = 9), Croatia (82.8 ± 26.6 kg kg^−1^, median = 68.2, n = 24), Nigeria (71.9 ± 11.4 kg kg^−1^, median = 71.5, n = 28), and USA (84.0 ± 61.7 kg kg^-1^, median = 68.2, n = 192) were relatively higher than the overall mean NUE.

**Figure 1:**
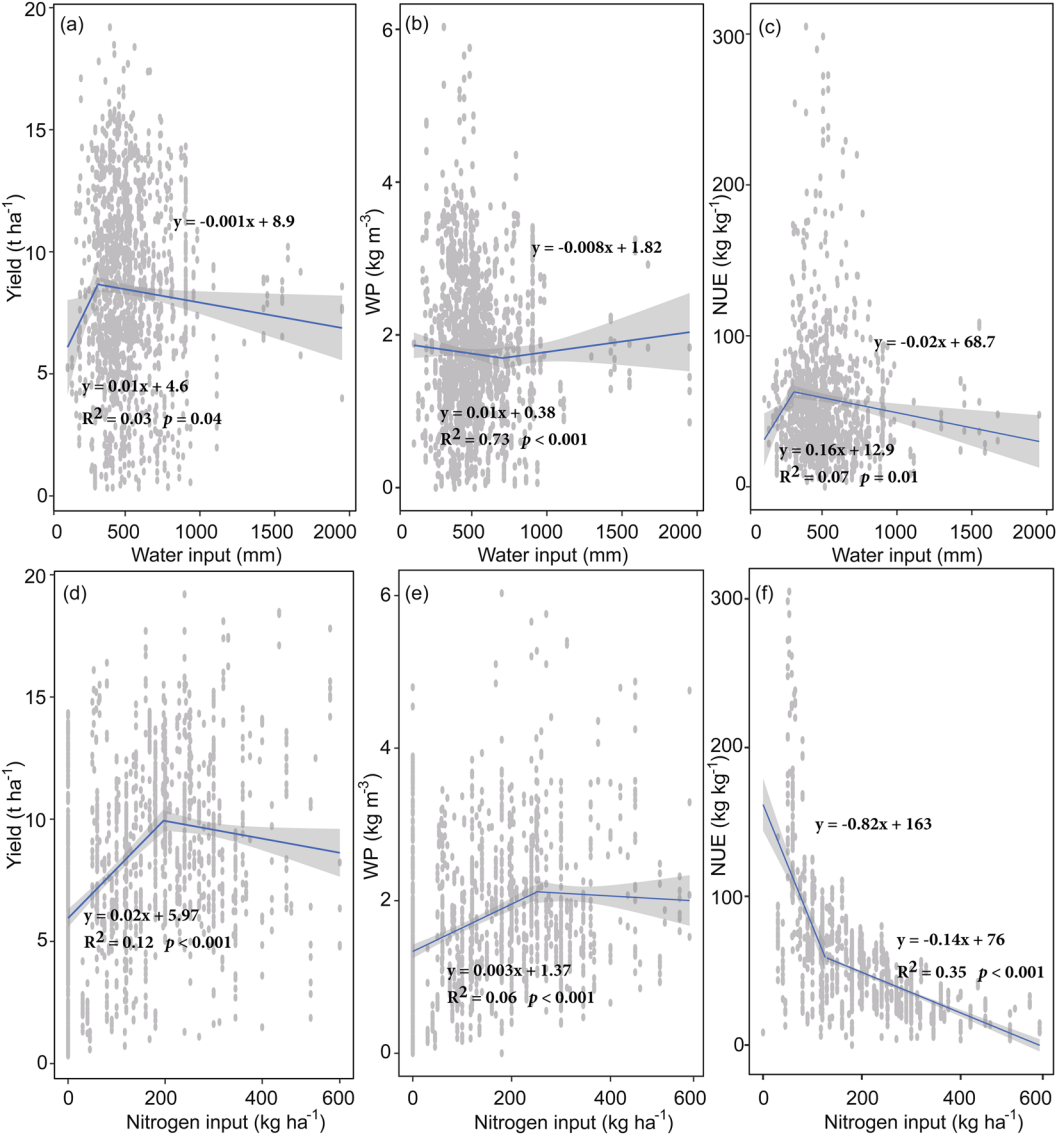
Relationships between total water input and maize (**a**) yield, (**b**) water productivity (WP), and (**c**) nitrogen use efficiency (NUE), and relationships between total nitrogen input and (**d**) yield, (**e**) water productivity (WP), and f) nitrogen use efficiency (NUE). Data points were split into two parts according to a breakpoint. The breakpoint between the two lines of water input is 314.4, 708.9, and 311 mm for yield, WP, and NUE, respectively. The breakpoint between the two lines of nitrogen is 249.9, 127.5, and 196 kg ha^−1^ for yield, WP, and NUE, respectively. Linear regressions were made for the sub-datasets accordingly, using the ordinary least square method. Grey areas indicate 95% confidence intervals. Note the different scales among the graphs.

Pooling data from all countries showed maize yield increased linearly with increasing water input, with 3% of the variation in yield explained when the total water input was less than 314 mm (*P* < 0.001, Fig. 1a), however, further water input actually declined maize yield (*P* = 0.04) with 3% of the variation explained. WP decreased linearly with increasing water input with 73% of the variation in WP explained when the total water input was higher than 709 mm (*P* < 0.001, Fig. 1b), otherwise, WP increased linearly with increasing water input, with 3% of the variation in WP explained (*P* < 0.001). Conversely, NUE increased linearly with increasing water input, with 7% of the variation in NUE explained when the total water input was less than 311 mm (*P* < 0.001, Fig. 1c), NUE declined linearly with increasing water input, with 1% of the variation in NUE explained.

Maize yield increased linearly with increasing N input, with 12% of the variation in grain yield explained when the total N input was less than 250 kg ha^−1^ (*P* < 0.001, Fig. 1d), and further N input had no effect on maize yield. Similarly, WP increased linearly with increasing N input with 6% of the variation in WP explained when the total N input was less than 128 kg ha^−1^ (*P* = 0.06, Fig. 1e), afterwards, WP had no response with increasing N input. Conversely, NUE declined linearly with increasing N input, with 17% of the variation in NUE explained when the total N input was less than 196 kg ha^−1^ (*P* < 0.001, Fig. 1f), then NUE declined slightly with increasing N input and 35% of the variation in NUE explained (*P* < 0.001).

The mixed-effects model suggested that maize yield was positively related to water or N inputs and (*P* < 0.001, Table 1). However, interactive effects (water × N inputs) had no effects on maize yield (*P* = 0.80). Also, planting density had a positive effect on maize yield (*P* < 0.001). Mean annual temperature suggested negative effects on yield (*P* < 0.001).

**Table 1:** Mixed-effects model of the effects of mean annual temperature (MAT), maize planting density (number of plants per hectare, PPH), initial soil organic carbon (SOC), and water (W) and nitrogen (N) input on yield, water productivity (WP) and N use efficiency (NUE).

Dependent variable	Model parameter	Value	Std. error	df	*t*-value	*p*-value
Yield (t ha^−1^)	α (intercept)	4.22	1.45	901	2.91	**0.004**
	β_1_ (MAT)	−0.25	0.05	35	−4.45	**<0.001**
	β_2_ (PPH)	<0.001	<0.001	901	3.81	**<0.001**
	β_3_(SOC)	0.01	0.06	901	0.10	0.92
	β_4_ (W)	0.01	<0.001	901	6.62	**<0.001**
	β_5_ (N)	0.01	0.002	901	5.10	**<0.001**
	β_5_ (W × N)	<0.001	<0.001	901	0.25	0.80
WP (kg m^−3^)	α (intercept)	2.30	0.36	913	6.38	**<0.001**
	β_1_ (MAT)	−0.05	0.01	36	−4.04	**<0.001**
	β_2_ (PPH)	<0.001	<0.001	913	2.32	**0.02**
	β_3_(SOC)	−0.01	0.01	913	−0.94	0.35
	β_4_ (W)	−1E-03	<0.001	913	−7.22	**<0.001**
	β_5_ (N)	0.004	<0.001	913	8.77	**<0.001**
	β_5_ (W × N)	−5E-06	<0.001	913	−5.18	**<0.001**
NUE (kg kg^−1^)	α (intercept)	105.2	19.07	703	5.51	**<0.001**
	β_1_ (MAT)	−2.53	0.67	35	−3.77	**<0.001**
	β_2_ (PPH)	<0.001	<0.001	703	2.48	**0.01**
	β_3_(SOC)	−1.66	0.72	703	−2.31	**0.02**
	β_4_ (W)	0.05	0.01	703	4.42	**<0.001**
	β_5_ (N)	−0.24	0.03	703	−7.24	**<0.001**
	β_5_ (W × N)	−1E-04	<0.001	703	−2.19	**0.03**

Bold signifies *p* < 0.05.

Water productivity was negatively related to MAT and water inputs (*P* < 0.001), and NUE was negatively related to MAT and N inputs (*P* < 0.001). Both WP (*P* = 0.02) and NUE (*P* = 0.01) were positively related to PPH. The interaction of water and N inputs negatively affected both WP (*P* < 0.001) and NUE (*P* = 0.03, Table 1). Initial SOC was negatively related to NUE (*P* = 0.02) but not to WP (*P* = 0.35).

### Input of water and N affects maize yield, WP and NUE

**Figure 2:**
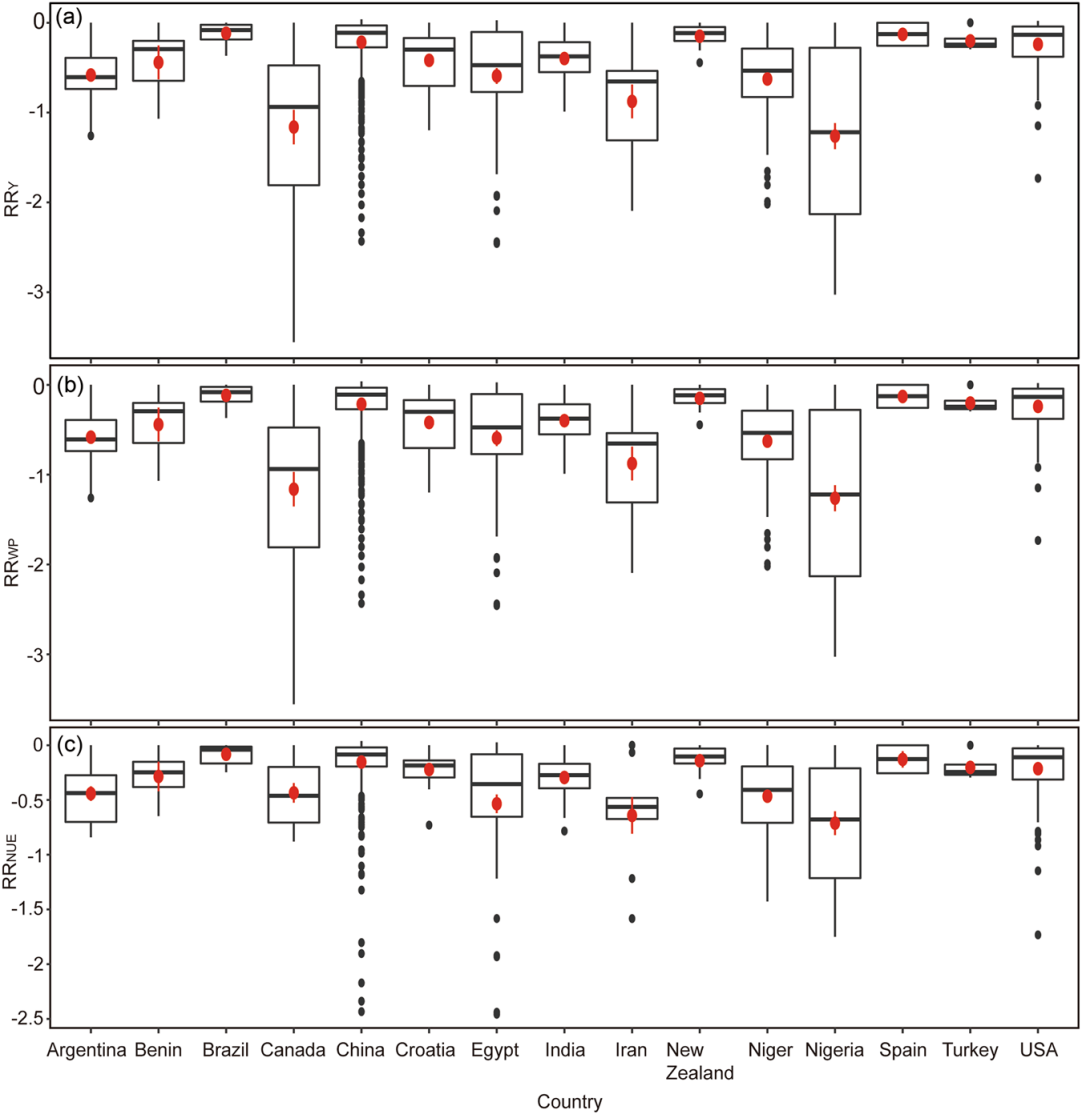
An overview of the response ratio of maize (**a**) yield (*RR*_Y_), (**b**) water productivity (*RR*_WP_), and (**c**) nitrogen use efficiency (*RR*_NUE_) over the main maize producing countries in the world. Response ratio stands for effect size between the treatment and control and is calculated using Eqs. 2–4. The horizontal line and the red dot (with standard deviation) indicate the median and average values; the limits of the boxes represent the 25 and 75 percentile (lower and upper limit, respectively); and vertical bars represent the 5 and 95 percentile. Note the different scales among the graphs.

*RR*_Y_, *RR*_WP_, and *RR*_NUE_ varied widely in Canada, Egypt, Iran, Niger, and Nigeria (Fig. 2). In contrast, *RR*_Y_, *RR*_WP_, and *RR*_NUE_ in Brazil, Croatia, Australia, and Turkey were relatively smaller than in other countries.

**Figure 3:**
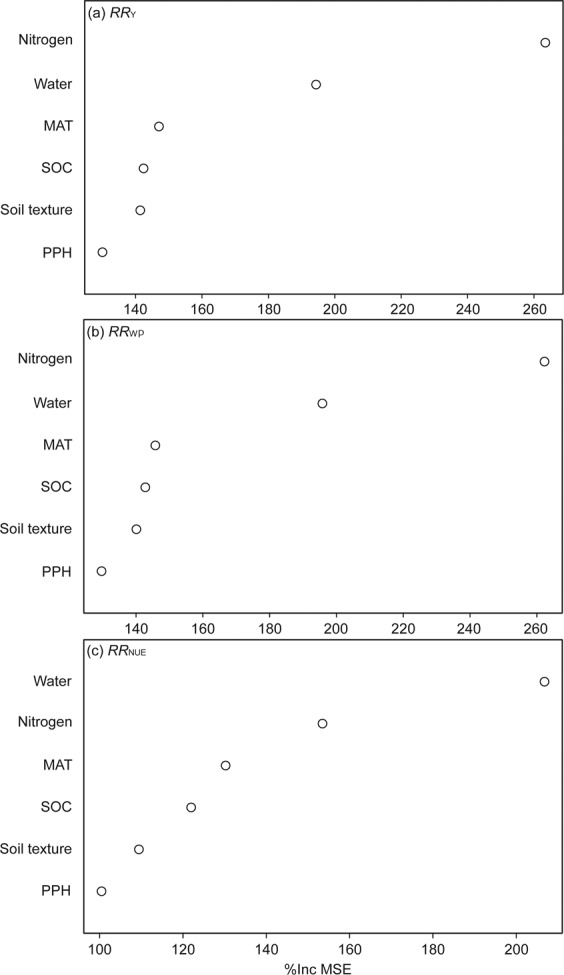
Increase in mean square error (IncMSE%) of variables on impacting the response ratio of (**a**) yield (*RR*_Y_), (**b**) water productivity (*RR*_WP_) and (**c**) nitrogen use efficiency (*RR*_NUE_). Response ratio stands for effect size between the treatment and control and is calculated using Eqs. 2–4. Higher IncMSE% means more important. Random forest analysis was used. Variables include initial soil organic carbon (SOC) concentration, mean annual temperature (MAT), total nitrogen input (Nitrogen), total water input (Water), soil texture, and maize planting density (number of plants per hectare, PPH). The out-of-bag error of a, b, and c were 24.1, 23.7 and 32.8%, respectively. Note the different scales among the graphs.

The importance of variables impacting *RR*_Y_, *RR*_WP_ and *RR*_NUE_ are displayed in Fig. 3. Water and N input were the most important factors affecting the gaps between achieved and attainable maize yield, WP, and NUE, and then followed by variables MAT, initial SOC, and soil texture. While PPH played a less important role in influencing *RR*_Y_, *RR*_WP_ and *RR*_NUE_.

**Figure 4:**
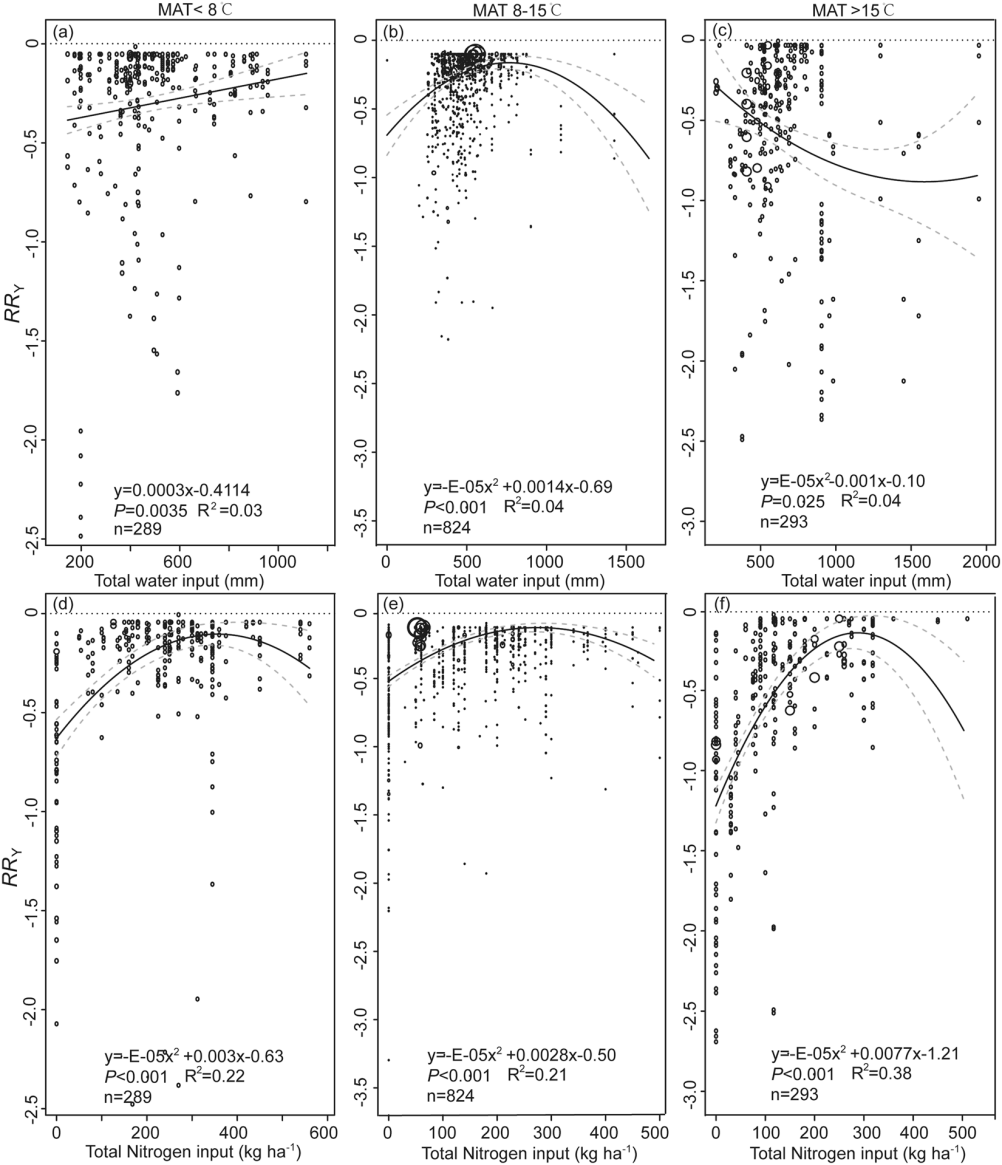
Scatter plot of response ratio of yield (*RR*_Y_) to water input when mean annual temperature (MAT) is (**a**) <8 °C, (**b**) 8–15 °C, or (**c**) >15 °C; to nitrogen input when MAT is (**d**) <8 °C, (**e**) 8–15 °C, or (**f**) >15 °C. Response ratio stands for effect size between the treatment and control and is calculated using Eq. 2. The solid black line represents the weighted regression line based on variance-weighted least squares. The grey line shows the 95% CI around the regression line. The circles indicate the response ratio of each observation. The circle size is proportional to the precision of the response ratio. Note the different scales among the graphs.

**Figure 5:**
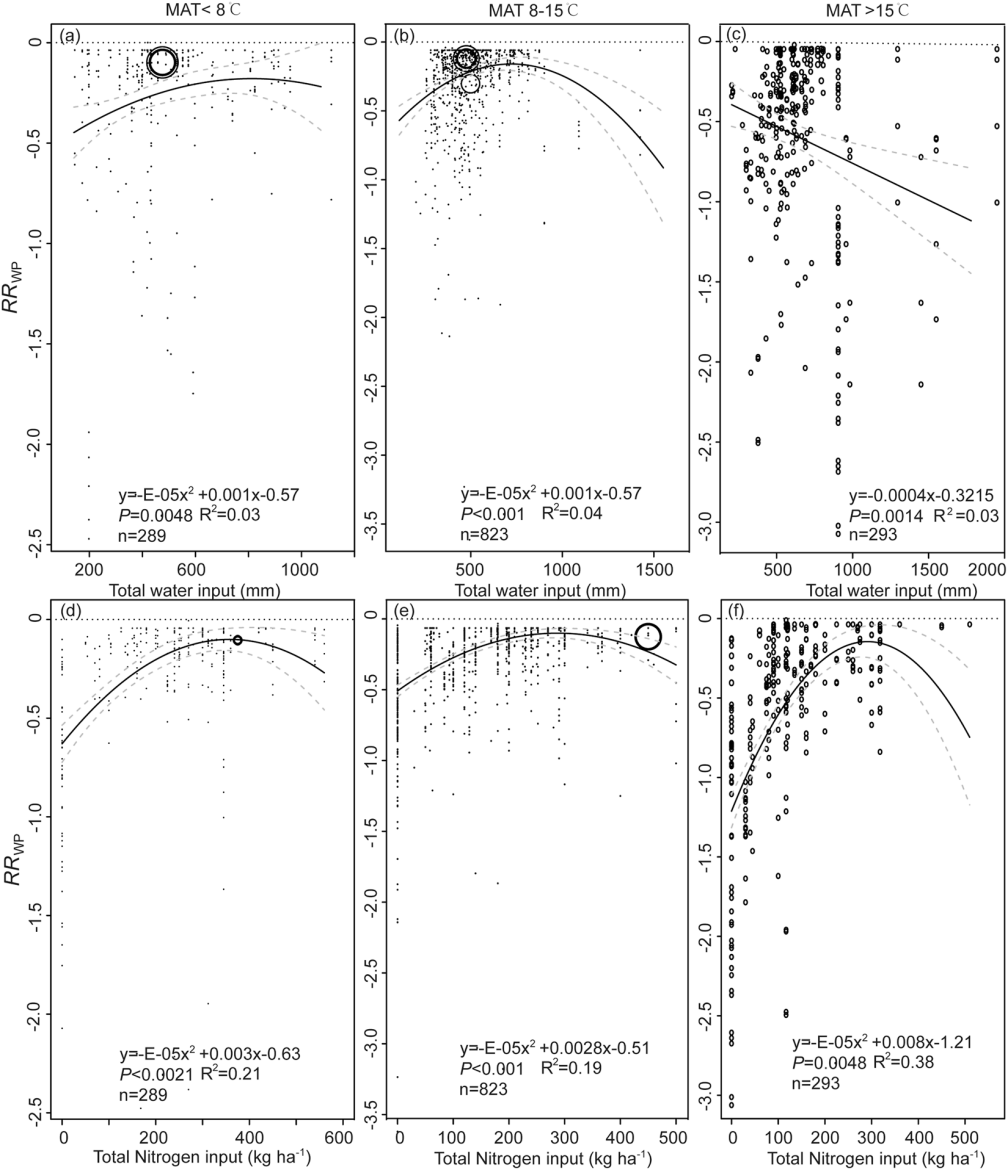
Scatter plot of response ratio of water productivity (*RR*_WP_) to water input when mean annual temperature (MAT) is (**a**) <8 °C, (**b**) 8–15 °C, or (**c**) >15 °C; to nitrogen input when MAT is (**d**) <8 °C, (**e**) 8–15 °C, or (**f**) >15 °C. Response ratio stands for effect size between the treatment and control and is calculated using Eq. 3. The solid black line represents the weighted regression line based on variance-weighted least squares. The grey line shows the 95% CI around the regression line. The circles indicate the response ratio of each observation. The circle size is proportional to the precision of the response ratio. Note the different scales among the graphs.

**Figure 6:**
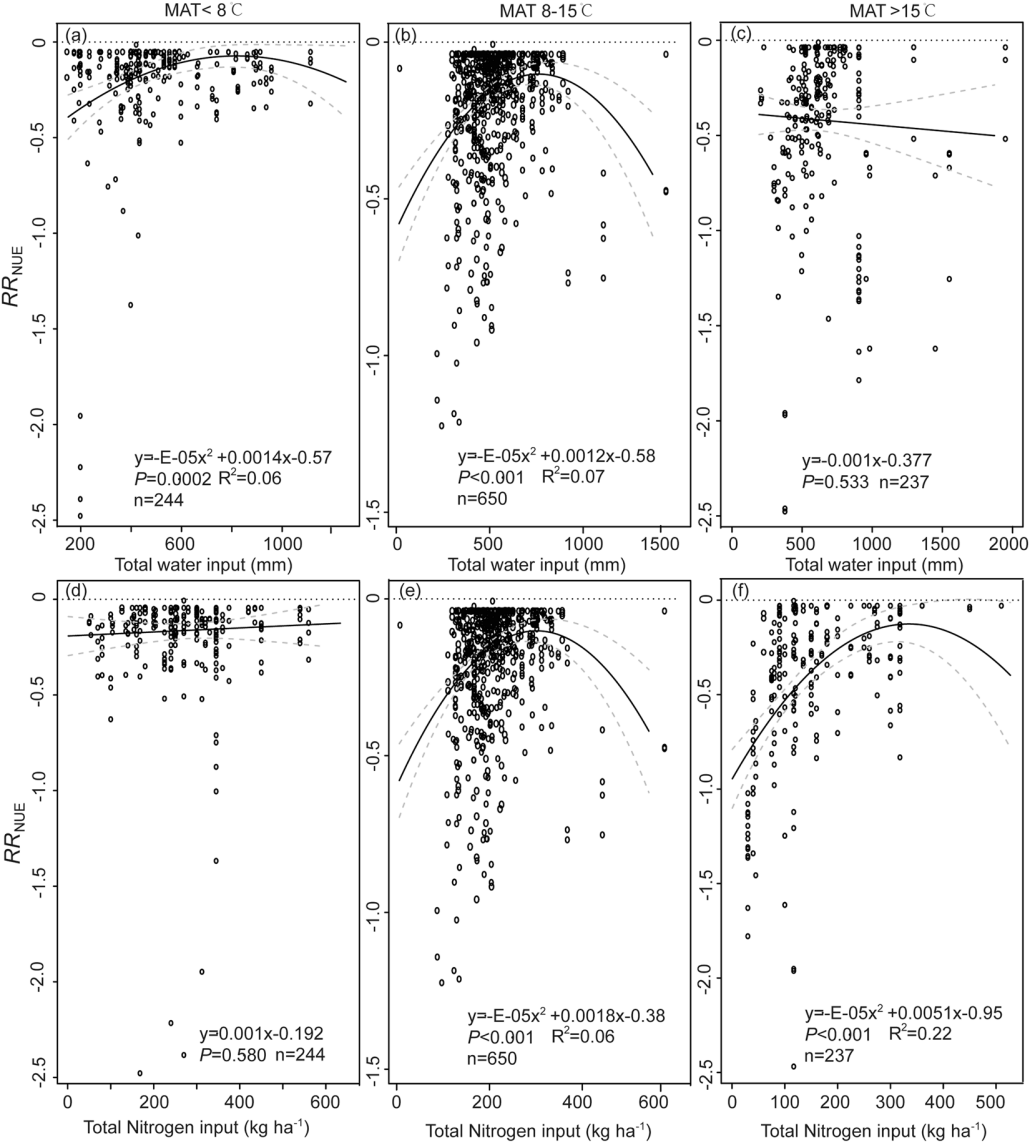
Scatter plot of response ratio of nitrogen use efficiency (*RR*_NUE_) to water input when mean annual temperature (MAT) is (**a**) <8 °C, (**b**) 8–15 °C, or (**c**) >15 °C; to nitrogen input when MAT is (**d**) <8 °C, (**e**) 8–15 °C, or (**f**) >15 °C. Response ratio stands for effect size between the treatment and control and is calculated using Eq. 4. The solid black line represents the weighted regression line based on variance-weighted least squares. The grey line shows the 95% CI around the regression line. The circles indicate the response ratio of each observation. The circle size is proportional to the precision of the response ratio. Note the different scales among the graphs.

Considering the importance of MAT in affecting maize yield, WP, and NUE (Table 1), and *RR*_Y_, *RR*_WP_ and *RR*_NUE_ (Fig. 3), the relationships between independent variables (i.e., water and N input) and dependent variables (i.e., *RR*_Y_, *RR*_WP_ and *RR*_NUE_, Figs. 4–6) were determined based on sub-dataset grouped by MAT. For regions where the MAT is less than 15 °C, there was a positive relationship between *RR*_Y_ and water input when the total water input quantity was less than 700 mm (Fig. 4a). There were negative relationships between *RR*_Y_, *RR*_WP_ and *RR*_NUE_ and water input when MAT was higher than 15 °C (Figs. 4–6c). However, some of these quadratic models indicated a poor-fitting to the entire data (R^2^ = 0.03–0.07).

The *RR*_Y_, *RR*_WP_ and *RR*_NUE_ increased with total N input when the N application rate was less than 300 kg kg^−1^ (Figs. 4–5d–f), with exception of NUE when MAT is less than 8 °C. Some of these quadratic equations explained a great amount of variation of the regressed data, and R^2^ ranged from 0.19 to 0.38.

**Figure 7:**
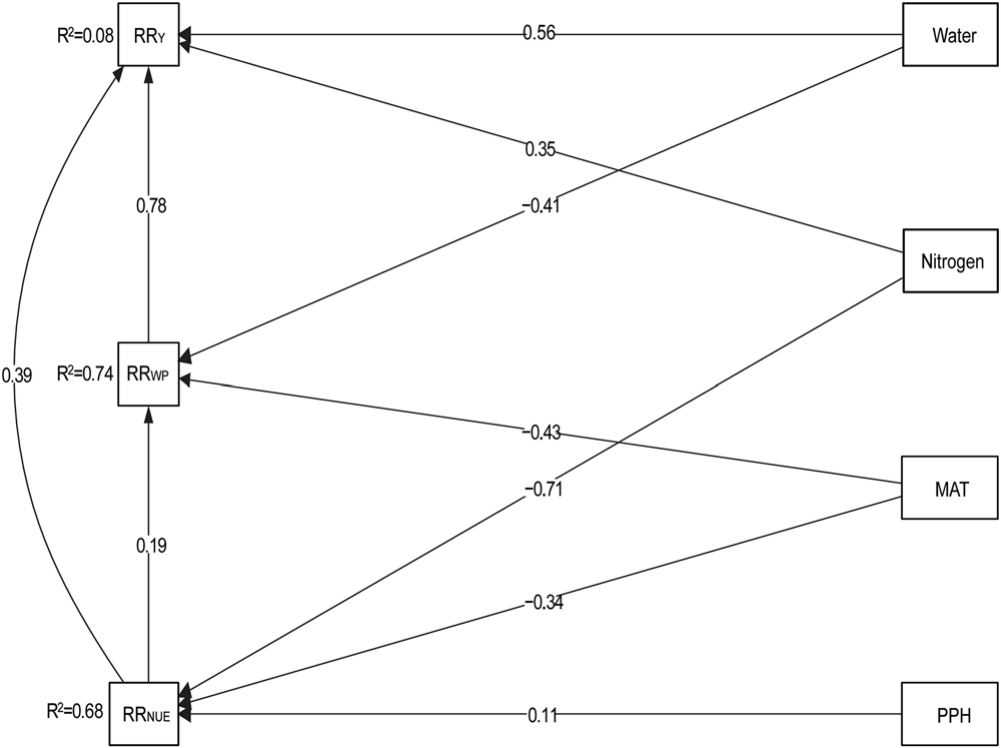
Structural equation model showing the direct and indirect effects of environmental and management conditions on the response ratio of yield (*RR*_Y_), water productivity (*RR*_WP_) and nitrogen use efficiency (*RR*_NUE_). Response ratio stands for effect size between the treatment and control and is calculated using Eqs. 2–4. Numbers at arrows are standardized path coefficients. Numbers next to boxes indicate the variance explained by the model (R^2^). MAT, mean annual temperature; PPH, maize planting density (number of plants per hectare). Water, total water input; Nitrogen, total nitrogen inputs. *χ*^2^ = 0.21; GFI = 0.998, AIC = 246.9, RMSEA = 0.05. The quality of fitting was assessed using *χ*^2^-test, the goodness of fit (GIF) and root mean squared error of approximation (RMSEA) indices, and Akaike information criterion (AIC) values, see details at section.

The final SEM model highlighted the importance of management practices (i.e., water and nitrogen input, and PPH) and climate factor (MAT) on improving *RR*_Y_, *RR*_WP_, and *RR*_NUE_ (Fig. 7). However, water and nitrogen inputs had an opposite effect on *RR*_Y_ and *RR*_WP_/*RR*_NUE_, and *RR*_WP_ showed the highest positive effect on *RR*_Y_.

## Discussion

There are a number of mechanisms illustrating the application of water and/or N could affect plants growth^17,18^. Crop NUE could be largely controlled by soil water availability by regulating several important processes in the N cycle and affecting N availability and plants uptake^10^. Hence, a slight increase at the below-threshold water level leads to an increase in yield and NUE, and above-threshold input significantly decreased yield and NUE (Fig. 1a,c). This increase, however, did not increase WP in our study, possibly because water demand of maize typically ranged from 500 to 800 mm^19^, the breakpoint of WP was 709 (Fig. 1b), and thus more water input is demanded to increase WP considering other water losses such as water evaporation and water runoff in the field. On the other hand, a reasonable supply of N can always enhance WP by promoting plant protein synthesis for supporting growth^7,20^. Likewise, a below-threshold N input increased yield and WP, while above threshold N input significantly decreased yield and WP (Fig. 1d,e). However, regression results only explained 3–73% of the variations, thereby indicating that there exist other environmental or managerial factors or interactions that affect productivity and NUE^16,17^. As indicated in this study, climate factor, e.g. MAT, probably play an important role in affecting maize production and resource use efficiency (Table 1). It has long been known that agricultural production is vulnerable to climate change, especially the temperature impacts^14^. Compiling extensive published results from global grid-based and local point-based models, statistical regressions, and field-warming experiments, Zhao, *et al*.^14^ reported that the reduction of yield for each degree Celsius increase in global mean temperature is largest for maize, -7.4 ± 4.5% per degree Celsius.

Temperature can be a universal limiting factor for crop yield^21^. Temperature increase, particularly night-time high temperature, most likely has negative impacts on cereal grain production^14,22^. Our study indicates that there is a negative correlation between MAT and yield (Table 1), and the lowest percentage change of yield took place when MAT was greater than 15 °C (Fig. 4a). Additionally, the increased water input decreased the *RR*_Y_ (Fig. 4c), *RR*_WP_ (Fig. 5c), and *RR*_NUE_ (Fig. 6c) when MAT was >15 °C. Using boundary-function analysis, a study in the Argentine Pampas region, using data from 30 stations, found that the highest maize yield was observed at moderate MAT, 21.8–23.5 °C^23^, which agrees with our findings. Maize is a typical C_4_ plant, however, high MAT leads to physiological stresses resulting in leaf curling, stomatal closure, reduction of CO_2_ assimilation rate, and shrinkage of the length of the growing cycle^24^. High temperature, particularly night-time high temperature, could increase crop respiration costs while shortening the grain-fill duration^25^. In addition, increased temperature enhances soil evaporation and thus water deficits^14,26^, eventually decreasing maize yields. A study compiling extensive published results from field warming experiments showed that MAT (21–29 °C) negatively affected crop yield of wheat, rice, maize, and soybean at the global scale^14^, and loss of yield due to the increase of MAT was the highest for maize. More importantly, we suspect that the MAT threshold might be 15 °C^9^ for maize production. This number is lower than the threshold value identified by a previous study^11^: 18 °C, which is the optimal temperature for phosphoenolpyruvate carboxylase, known as the primary enzyme in charge of carbon fixation processes in C_4_ photosynthetic pathway.

Additionally, despite research indicating that high-temperature stresses could be easily confounded with moisture stress^27^, we found that the increased water input did reduce the gaps between actual and attainable yield in frigid areas (Fig. 4a) but not in thermic temperature areas (Fig. 4c). This is likely that MAP is generally higher, possibly with concurrently low elevation, in thermic temperature areas than that in frigid areas^28^. In other words, water input from irrigation is the limiting factor of maize yield in low MAP areas instead of high MAP areas^29^. Varietal changes can be made to assist to preserve the pre-flowering length^30^ against the shortening effect of warming and compensate for the negative effects of changes in climate (i.e. temperature and radiation)^31^.

The quadratic meta-regression results indicated that the threshold of efficiently using water and N input for global maize production was around 700 kg m^−3^ and 300 kg kg^−1^, respectively, in most cases (Figs. 4–6). However, both inputs of water and N were much greater than the average values in most regions^9^. These partly explain that the achieved yield, WP and NUE were significantly lower than the optimal values. While adjusting water or N input independently would only optimize productivity and NUE to a certain extent (Fig. 1). High input of either water or N may postpone the phenological development of maize^10^ due to prolonged vegetative growth stage and delayed maturity. In contrast, treatments with a low irrigation level and reduced N dose could also result in significantly shortened vegetative growth stage and early maturity. Thus, disregarding the MAT subgroup, the responses of yield (Fig. 4b–f), WP (Fig. 5a–b,d–f)), or NUE (Fig. 6a,b,e,f) to the input of water or N typically yield a quadratic relationship. Considering the increase in global mean temperature, our results are critical in assessing future climate impacts on global maize production using models or field-warming experiments. Hence, adaptation strategies, possibly combining earlier sowing dates and selection of longer-season maize cultivars, could be a general approach to optimize yield, and water and N utilization efficiency on a global scale.

Row spacing greatly affects yield per unit land area regardless of crop species^32,33^. However, it is less important than other factors, such as water and N management, or climate conditions. Using a similar dataset, previous research has reported that the overall mean PPH was 6.9 ± 1.5 plants m^−2^ ^9^, and Testa, *et al*.^32^ suggested that planting density between 7.5 and 10.5 plants m^−2^ should be appropriate under irrigation and fertilizer conditions. Therefore, if PPH was managed properly in studies used in the current research, then future research should pay more attention to other factors to improve maize yield, and resource use efficiencies.

Due to data quality issues, criteria of data selection used in the current study does not include the interactions with other critical managerial practices, such as mulching and cover cropping practices. Also, many resource input and climatic data used in this study (i.e., nitrogen and/or water input, growing season temperatures) was not stratified according to production phase or developmental stages. Additionally, almost 50% studies used in this study were in China. Thus, interpretation should be made with caution. It is also noteworthy to mention that the Mainland China features a broad array of soil types and climate conditions, thus, the results can still provide a broader scope of interpretation.

## Conclusions

Input of either water or N has a significantly positive impact on yield, WP, or NUE of maize. The current study quantified critical thresholds (input of water and N) and MAT impacting production and resource use efficiency for maize production. We found water input and *RR*_WP_ were more important than N input and *RR*_NUE_ in improving *RR*_Y_. Moreover, to achieve the highest yield by optimizing WP and NUE, a MAT value around 15 °C was observed. The results, threshold values (i.e., levels of water and N) and key drivers (i.e., MAT), will be helpful for large-scale modelling effort and improving water and/or N management strategies for maize production in the future. In the meantime, site-specific studies are warranted to verify results of the current study in the future.

## Materials and methods

### Data collection

This study used the dataset from our previously published meta-analysis^9^ with identical data selection criteria and filtering methods. The final analysis was based on 1436 yield observations from 83 studies conducted in 15 countries (Fig. S1). Afterwards, information related to soil property (soil texture, initial soil organic carbon concentration (SOC)), climatic conditions, mean annual precipitation (MAP), and mean annual temperature (MAT), and maize planting density (number of plants per hectare, PPH) were collated from each study. Particularly, the whole dataset was organized according to MAT [frigid (<8 °C), mesic (8–15 °C), or thermic (>15 °C)] MAT regimes^34^.

### Definitions and data analysis

Water productivity (WP, kg m^–3^) was calculated as grain yield (Y, kg ha^−1^) divided by the total amount of water (W, rainfall + irrigation, mm) input in crop production^20^. Nitrogen use efficiency (kg kg^−1^) was calculated as: NUE = Y/N, where N is the total amount of N fertilizer applied (kg ha^−1^).

### Estimating the mean effects of environmental or managerial factors

The effects of water and N input, MAT, PPH, initial SOC and on maize yield, WP and NUE were assessed using a mixed-effect model via the R package “nlme^35^”, using soil texture as a random effect factor:

1 $${\rm{Y}}={\rm{\alpha }}+{\beta }_{1}\times W+{\beta }_{2}\times N+{\beta }_{3}\times MAT+{\beta }_{4}\times PPH+{\beta }_{5}\times SOC+{\beta }_{6}\times W\times N+error$$

where Y is yield (t ha^−1^), *β*_1–6_ represent variable effects, W represents total water (rainfall + irrigation, mm) input, N represents the total fertilizer N input (kg ha^−1^), W × N indicates the interaction of water and N input, and error is the residual that was not explained by the independent variables. All independent variables are numeric. Similar analyses were also conducted for WP and NUE.

Additionally, linear regression was conducted to illustrate the effect of water and N input on yield, WP, and NUE. While yield, WP, and NUE were plotted as a function of water and N inputs and fit with a broken-stick linear regression model with a critical limiting value, which can be described with two straight lines that intersect at a threshold value^36^ using the ‘segmented’ package^37^. The broken-stick linear regression model was mainly used to identify threshold values at which the effects on response variables reverse as increasing water or N inputs; instead of identifying linear models.

### Estimating the factors affecting yield, WP, and NUE

Optimal water and N inputs were defined as ‘the least input levels that produced the highest reported yield in a specific year of a study’^9,16^. The effect size of below-optimal and above-optimal water and N inputs on yield was calculated using the natural logarithm of the response ratio (*RR*)^38^:

2 $$R{R}_{{\rm{Y}}}=\,\mathrm{ln}\left(\frac{{Y}_{obs}}{{Y}_{ref}}\right)=\,\mathrm{ln}({Y}_{obs})-\,\mathrm{ln}({Y}_{ref})$$

where *Y*_obs_ is the observed yield in a specific year of a study, and *Y*_ref_ is the highest reported yield (reference yield) in a specific year of a study. Likewise, the intensity of below-optimal and above-optimal water and N input on WP and NUE were estimated for each study as:

3 $$R{R}_{{\rm{WP}}}=\,\mathrm{ln}\left(\frac{W{P}_{obs}}{W{P}_{ref}}\right)=\,\mathrm{ln}(W{P}_{obs})-\,\mathrm{ln}(W{P}_{ref})$$

4 $$R{R}_{{\rm{NUE}}}=\,\mathrm{ln}\left(\frac{NU{E}_{obs}}{NU{E}_{ref}}\right)=\,\mathrm{ln}(NU{E}_{obs})-\,\mathrm{ln}(NU{E}_{ref})$$

where *WP*_obs_ and *NU*E_obs_ are the observed WP and NUE in a specific year of a study, and *WP*_ref_ and *NUE*_ref_ are the WP and NUE associated with optimal water and N inputs of a specific year of a study.

Using ‘randomForest’ package^39^, the importance of variables on *RR*_Y_, *RR*_WP_, and *RR*_NUE_ was estimated. This nonparametric method allowed for the consideration of all observations for assessing the relationship of predictors to the change in response to *RR*_Y_, *RR*_WP_, and *RR*_NUE_ and various environmental (initial soil organic carbon concentrations, soil texture, and MAT) and management (water and N inputs, and PPH) factors.

Using the ‘metafor’ package^40^ with the restricted maximum likelihood (RMLE) and the Knapp and Hartung (KH) adjustment, a meta-regression analysis was performed to investigate the linear associations between the *RR*_Y_, *RR*_WP_, *RR*_NUE_ to water or nitrogen input.

Finally, structural equation model (SEM) was used to disentangle indirect and direct effects of climate (MAT), and management practices (water and N inputs, and PPH) on the *RR*_Y_, *RR*_WP_, and *RR*_NUE_ using the ‘lavaan’ package^41^. All data were normalized (to ensure that the data fit a standard normal distribution with a mean of 0 and a standard deviation of 1), and an a priori regression analyses was established based on the known effects and relationships among the variables^42^. Rather than multiple regressions, SEM can be used to model multiple causal and/or interactive interrelationships among variables, which results in multiple explanatory and/or response variables in one model^42^. Data were fitted to the models using the maximum likelihood estimation method. The quality of fitting was assessed using the *χ*^2^-test, goodness of fit (GIF) index, and root mean squared error of approximation (RMSEA) indices. An accurate model should have no significant differences between the observed and simulated data evaluated using the χ^2^-test (*p* > 0.05). Additionally, the model should also provide high GIF (>0.9), low RMSEA (<0.08), and low Akaike information criterion (AIC) values^43^.

## Supplementary information

Supplementary information.

## References

[1] Alexandratos, N. & Bruinsma, J. *World agriculture towards 2030/2050: the 2012 revision*, http://large.stanford.edu/3174B40D-38BB-4666-9BF0-B83C5A13CC3E/FinalDownload/DownloadId-83BF020A7CB21638499FB8BD241C3D52/3174B40D-38BB-4666-9BF0-B83C5A13CC3E/courses/2014/ph240/yuan2/docs/ap106e.pdf (2012).

[2] Tilman D, Balzer C, Hill J, Befort BL (2011). Global food demand and the sustainable intensification of agriculture. Proc Natl Acad Sci USA.

[3] Foley, J. *It’s time to rethink America’s corn system*, https://www.scientificamerican.com/article/time-to-rethink-corn/ (2013).

[4] Mekonnen MM, Hoekstra AY (2014). Water footprint benchmarks for crop production: A first global assessment. Ecological Indicators.

[5] Raun WR, Johnson GV (1999). Improving nitrogen use efficiency for cereal production. Agron J.

[6] Rosegrant MW, Ringler C, Zhu T (2009). Water for agriculture: maintaining food security under growing scarcity. Annul Rev Env Resour.

[7] Zwart SJ, Bastiaanssen WGM (2004). Review of measured crop water productivity values for irrigated wheat. rice, cotton and maize. Agric Water Manag.

[8] Ladha JK (2016). Global nitrogen budgets in cereals: A 50-year assessment for maize, rice, and wheat production systems. Sci Rep.

[9] Li Y (2019). A global synthesis of the effect of water and nitrogen input on maize (*Zea mays*) yield, water productivity and nitrogen use efficiency. Agric For Meteorol.

[10] Ashraf U (2016). Maize growth, yield formation and water-nitrogen usage in response to varied irrigation and nitrogen supply under semi-arid climate. Turk J Field Crops.

[11] Kingston-Smith AH, Harbinson J, Foyer CH (1999). Acclimation of photosynthesis, H_2_O_2_ content and antioxidants in maize (*Zea mays*) grown at sub-optimal temperatures. Plant Cell Environ.

[12] Allen DJ, Ort DR (2001). Impacts of chilling temperatures on photosynthesis in warm-climate plants. Trends Plant Sci.

[13] Wilson JH, Clowes MSJ, Allison JCS (1973). Growth and yield of maize at different altitudes in Rhodesia. Ann Appl Biol.

[14] Zhao C (2017). Temperature increase reduces global yields of major crops in four independent estimates. Proc Natl Acad Sci USA.

[15] Tollenaar M, Wu J (1999). Yield improvement in temperate maize is attributable to greater stress tolerance. Crop Sci.

[16] Qin W, Assinck FBT, Heinen M, Oenema O (2016). Water and nitrogen use efficiencies in citrus production: A meta-analysis. Agric Ecosyst Environ.

[17] Hernández M (2015). Maize water use efficiency and evapotranspiration response to N supply under contrasting soil water availability. Field Crops Res.

[18] Bennett JM, Mutti LSM, Rao PSC, Jones JW (1989). Interactive effects of nitrogen and water stresses on biomass accumulation, nitrogen uptake, and seed yield of maize. Field Crops Res.

[19] Critchley, W., Siegert, K. & Chapman, C. *A manual for the design and construction of water harvesting schemes for plant production*, www.fao.org/docrep/u3160e/u3160e04.htm (1991).

[20] Ragab, R. *A note on Water use efficiency and water productivity*, http://www.water4crops.org/wp-content/uploads/2014/08/RR_Water-use-efficiency-and-water-productivity.pdf (2012).

[21] van Ittersum MK, Rabbinge R (1997). Concepts in production ecology for analysis and quantification of agricultural input-output combinations. Field Crops Res.

[22] Muchow RC, Sinclair TR, Bennett JM (1990). Temperature and solar radiation effects on potential maize yield across locations. Agron J.

[23] Andrade JF, Satorre EH (2015). Single and double crop systems in the Argentine Pampas: Environmental determinants of annual grain yield. Field Crops Res.

[24] Wang X (2014). Divergence of climate impacts on maize yield in Northeast China. Agric Ecosyst Environ.

[25] Peng S (2004). Rice yields decline with higher night temperature from global warming. Proc Natl Acad Sci USA.

[26] Badu-Apraku B, Hunter RB, Tollenaar M (1983). Effect of temperature during grain filling on whole plant and grain yield in maize (*Zea mays* L.). Can J Plant Sci.

[27] Lobell DB (2014). Greater sensitivity to drought accompanies maize yield increase in the US Midwest. Science.

[28] Johnson BG, Verburg PSJ, Arnone JA (2014). Effects of climate and vegetation on soil nutrients and chemistry in the Great Basin studied along a latitudinal-elevational climate gradient. Plant Soil.

[29] Zhang G (2017). Optimizing water use efficiency and economic return of super high yield spring maize under drip irrigation and plastic mulching in arid areas of China. Field Crops Res.

[30] Egli DB (2011). Time and the productivity of agronomic crops and cropping systems. Agron J.

[31] Liu Y, Wang E, Yang X, Wang J (2010). Contributions of climatic and crop varietal changes to crop production in the North China Plain, since 1980s. Global `Change Biol.

[32] Testa G, Reyneri A, Blandino M (2016). Maize grain yield enhancement through high plant density cultivation with different inter-row and intra-row spacings. Eur J Agron.

[33] Casal JJ, Deregibus VA, SÁNchez RA (1985). Variations in tiller dynamics and morphology in Lolium multiflorum Lam. vegetative and reproductive plants as affected by differences in red/far-red irradiation. Ann Bot (Lond).

[34] Knorr M, Frey SD, Curtis PS (2005). Nitrogen additions and litter decomposition: A meta‐analysis. Ecology.

[35] Pinheiro, J., Bates, D., DebRoy, S. & Sarkar, D. *nlme: linear and nonlinear mixed effects models*. *R package version 3.1–117*, URL: http://cran.rproject.org/web/packages/nlme/index.html (2014).

[36] Sadras VO, Milroy SP (1996). Soil-water thresholds for the responses of leaf expansion and gas exchange: A review. Field Crops Res.

[37] Muggeo VM (2008). Segmented: an R package to fit regression models with broken-line relationships. R News.

[38] Hedges LV, Gurevitch J, Curtis PS (1999). The meta‐analysis of response ratios in experimental ecology. Ecology.

[39] Liaw, A. & Wiener, M. Classification and regression by randomForest. *R news* **2**, 18–22, https://www.r-project.org/doc/Rnews/Rnews_2002-3.pdf (2002).

[40] Viechtbauer W (2010). Conducting meta-analyses in R with the metafor package. J Stat Softw.

[41] Rosseel Y (2012). Lavaan: An R package for structural equation modeling. J Stat Softw.

[42] Grace, J. B. In *Structu*ral e*qu*ation *modeling and natural systems* (ed. James B. Grace) 324–349 (Cambridge University Press, 2006).

[43] Schermelleh-Engel K, Moosbrugger H, Müller H (2003). Evaluating the fit of structural equation models: Tests of significance and descriptive goodness-of-fit measures. MPR-online.

