# Supplementary material for: Determining effects of water and nitrogen input on maize (Zea mays) yield, water- and nitrogen-use efficiency: A global synthesis
Source: Sci Rep. 2020 Jun 16;10:9699. doi: 10.1038/s41598-020-66613-6 (PMC7297966; doi:10.1038/s41598-020-66613-6)
Supplement: Supplementary file 1 — Supplementary information. [file 41598_2020_66613_MOESM1_ESM.pdf]

Determining effects of water and nitrogen input on maize (*Zea mays*) yield, water- and nitrogen-use efficiency: A global synthesis

Yuan Li[a, b], Song Cui[c], Zhixin Zhang[d], Kezhang Zhuang[e], Zhennan Wang[a, *], Qingping Zhang[a, *]

[a] College of Agriculture and Forestry Science, Linyi University, Linyi 276000, P. R. China

[b] Biogeochemistry Research Group, Department of Environmental and Biological Sciences, University of Eastern Finland, PO Box 1627, Kuopio, Finland

[c] School of Agriculture, Middle Tennessee State University, Murfreesboro, TN 37132, USA

[d] College of Grassland Agriculture, Northwest A&F University, Yangling, 712100, P. R. China

[e] Linyi Agricultural Academy of Sciences, Linyi, Shandong 276012, P. R. China

[*]Corresponding author at College of Agriculture and Forestry Science, Linyi University, Linyi 276000, P. R. China

E-mail address: wangzn11@163.com (Z. Wang) and zhangqingping@lyu.edu.cn (Q. Zhang)

Supplementary figure captions

Fig. S1. The geographical coverage of the studies used in this study. Argentina (36 observations), Benin (5), Brazil (12), Canada (24), China (815), Croatia (36), Egypt (54), India (67), Iran (12), New Zealand (10), Niger (70), Nigeria (42), Spain (4), Turkey (6), and USA (244). The map was generated using ggplot2 (v. 3.3.0, [1]) and ggmap (v. 3.0.0, [2]) in RStudio (v. 1.2.5033, https://www.rstudio.com/).

Fig. S2. An overview of maize a) yields, b) water productivity (WP), and c) nitrogen use efficiency (NUE) over the main maize producing countries in the world. The horizontal line and the red dot (with standard deviation) indicate the median and average values; the limits of the boxes represent the 25 and 75 percentile (lower and upper limit, respectively); and vertical bars represent the 5 and 95 percentile. Grey lines indicate overall mean of maize yield, WP, and NUE, which was 8.3 ± 3.7 (mean ± SD) t ha^{-1}, 1.72 ± 0.96 kg m^{-3}, 55.7 ± 43.0 kg kg^{-1}, respectively. Note the different scales among the graphs.

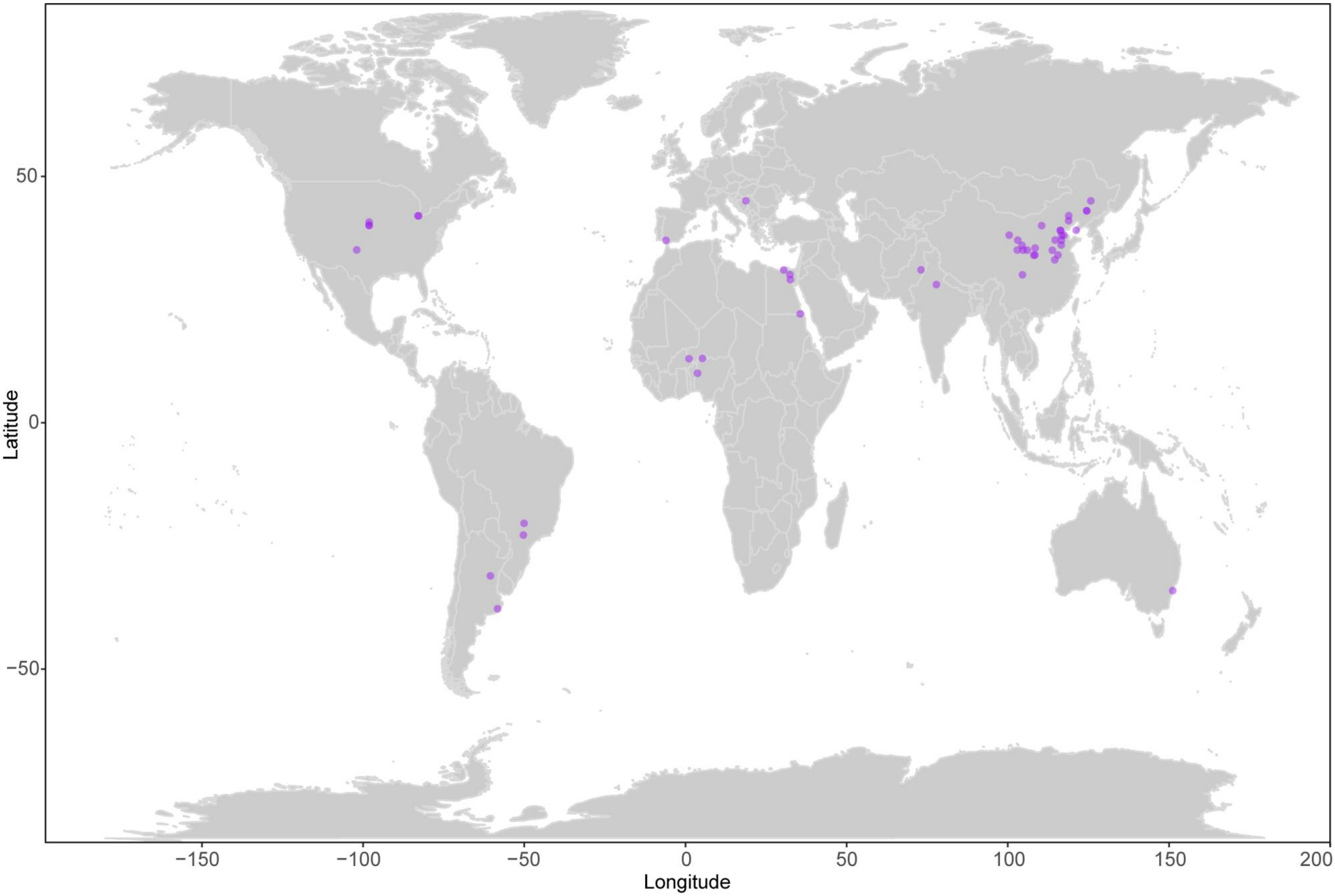

Figure S1

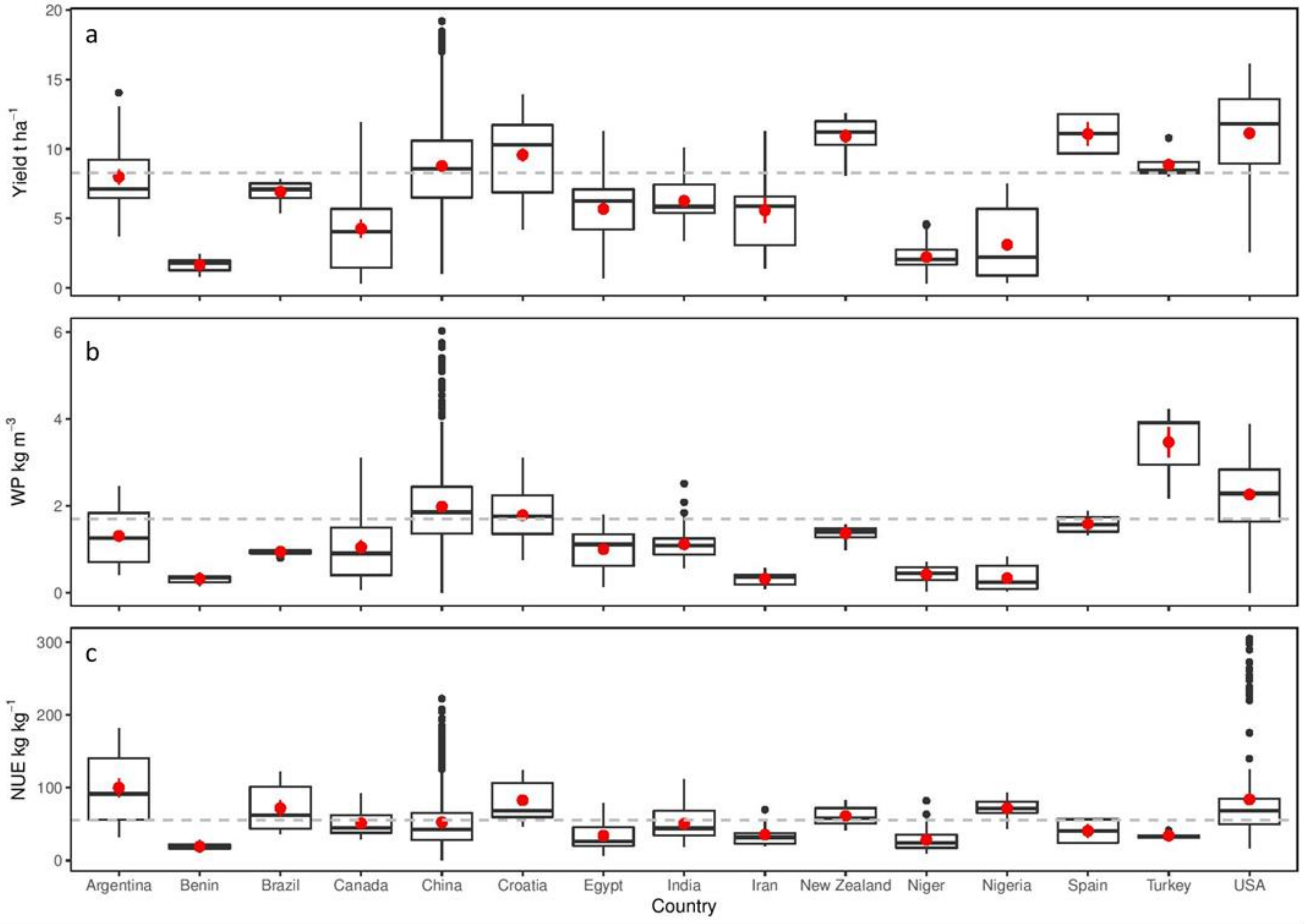
a
b
c
Yield t ha⁻¹
WP kg m⁻³
NUE kg kg⁻¹
0
5
10
15
20
0
2
4
6
0
100
200
300
Argentina
Benin
Brazil
Canada
China
Croatia
Egypt
India
Iran
New Zealand
Niger
Nigeria
Spain
Turkey
USA
Country

Figure S2

Reference

1 Wickham, H. *ggplot2: elegant graphics for data analysis*. (Springer, 2016).
2 Kahle, D. & Wickham, H. ggmap: Spatial Visualization with ggplot2. *The R journal* **5**, 144-161 (2013).
